# Characteristics and Outcomes of Elderly Patients Refused to ICU

**DOI:** 10.1155/2013/590837

**Published:** 2013-12-25

**Authors:** María-Consuelo Pintado, Patricia Villa, Natalia González-García, Jimena Luján, Rocío Molina, María Trascasa, Esther López-Ramos, Cristina Martínez, José-Andrés Cambronero, Raúl de Pablo

**Affiliations:** ^1^Intensive Care Unit, Hospital Universitario Príncipe de Asturias, Carretera Alcalá-Meco, s/n, Alcalá de Henares, 28805 Madrid, Spain; ^2^Palliative Care Unit, Hospital Universitario Príncipe de Asturias, Alcalá de Henares, 28805 Madrid, Spain; ^3^Department of Medicine, University of Alcalá, Alcalá de Henares, 28871 Madrid, Spain

## Abstract

*Background*. There are few data regarding the process of deciding which elderly patients are refused to ICU admission, their characteristics, and outcome. *Methods*. Prospective longitudinal observational cohort study. We included all consecutive patients older than 75 years, who were evaluated for admission to but were refused to treatment in ICU, during 18 months, with 12-month followup. We collected demographic data, ICU admission/refusal reasons, previous functional and cognitive status, comorbidity, severity of illness, and hospital and 12-month mortality. *Results*. 338 elderly patients were evaluated for ICU admission and 88 were refused to ICU (26%). Patients refused because they were “too ill to benefit” had more comorbidity and worse functional and mental situation than those admitted to ICU; there were no differences in illness severity. Hospital mortality rate of the whole study cohort was 36.3%, higher in patients “too ill to benefit” (55.6% versus 35.8%, *P* < 0.01), which also have higher 1-year mortality (73.7% versus 42.5%, *P* < 0.01). High comorbidity, low functional status, unavailable ICU beds, and age were associated with refusal decision on multivariate analysis. *Conclusions*. Prior functional status and comorbidity, not only the age or severity of illness, can help us more to make the right decision of admitting or refusing to ICU patients older than 75 years.

## 1. Introduction

There are no defined intensive care unit (ICU) admission criteria based on scientific evidence, so that decisions depend often on triaging doctor with marked variation in the rejection rates between different units [1–3]. With the increasing demand for critical care resources, often exceeding supply, its rationing is imposing [4]. This includes refusal of patients who are too ill or too well to benefit from critical care.

Several studies had shown that ICU refusal rates increase with increasing patient age, underlying disease, living in an institution, prior cognitive impairment, dependency, medical reason for admission, and bed availability [2–7].

Some authors have shown that outcomes of elderly patients after ICU admission are bad, with higher ICU mortality than younger patients, high 1-year mortality, and deterioration of functional autonomy and quality of life [3, 8, 9], supporting the idea of futility in the ICU admission of these patients. In contrast, other authors argue that patients should not be rejected solely because of age and consider that the most important determinant for ICU mortality is severity of illness, proposing that ICU practices should change [3–10].

The characteristics of elderly patients who were refused to ICU admission have been poorly analyzed. Garrouste-Orgeas et al. [9] described that medical patient, full unit, age > 85 years, and help needed for toileting were factors independently associated with refusal to ICU admission. However, in that study all refused patients were included in the logistic regression analysis, including patients too ill and too well to benefit from critical care.

Thus, we designed a prospective observational study to explore the outcome and characteristics of elderly patients refused to intensive care unit admission. The main objective of our study was to evaluate 1-year mortality of elderly patients refused to ICU. In addition, we also studied the characteristics of elderly patients referred to ICU admission, rates of refusal/admission to ICU and reasons motivating those decisions, factors associated to ICU refusal, hospital mortality, and number of rehospitalizations during the first-year followup.

## 2. Materials and Methods

This is a prospective longitudinal observational cohort study conducted in a 14-bed Medical and Surgical ICU at the University Hospital Principe de Asturias in Alcalá de Henares, Spain.

All consecutive patients older than 75 years, who were evaluated for admission to but were refused to treatment in ICU, during 18 months from December 2009 to May 2011, were included.

Decisions regarding admission were made by ICU staff physician, who followed usual admission criteria, without predefined eligibility or exclusion criteria for those patients.

At the time of evaluation the following data were collected: age, sex, reason for triage, admission diagnosis, admission/refusal to ICU, referral site (emergency room, operating/recovery room, or ward), prior functional status (estimated by the Barthel Index (BI) [11]), prior cognitive status (determined according to the Cruz Roja Mental Scale (CRMS) [12]), comorbidity (measured by the Charlson Comorbidity Index (CCI) [13]), and evaluation of severity of illness at time of triage by the Acute Physiology, Age, and Chronic Health Evaluation Score (APACHE II) [14] and Sepsis-Related Organ Failure Assessment (SOFA) [15]. We defined functional independence as a punctuation above 60 on BI [11] and severe mental incapacity as a punctuation over 3 on Cruz Roja Mental Scale [16]. We defined 3 levels of comorbidity according to the CCI [13]: low (score: 0 or 1), medium (score: 2), and high (score: 3 or over). APACHE II score [14] was calculated for each patient using the data obtained at the time of evaluation. A one-year prospective followup of all patients included in the study was performed, including records of hospital stay, hospital mortality, vital status at 1 year, and number of rehospitalizations during that time. Outcome data were obtained by medical record (BI, CRMS, and CCI scores are shown in Supplementary Material available online at http://dx.doi.org/10.1155/2013/590837).

We also recorded reasons for refusal of ICU admission at triage using one-choice items, which included patients “too well to benefit” (inappropriate referral—these patients would not be considered for ICU admission and should never even have been referred), patients “too ill” (patients who were too ill on which ICU care was considered futile or who, it was felt, would derive some benefit from ICU care but insufficient benefit to be accorded a high enough priority to meet the admission threshold) and patient/family refused ICU admission; in this last option patients were excluded from study. When the reason for refusal of ICU given was “too ill,” the triage physician recorded second reasons using multiple-choice items, which included patient “too old,” high grade of comorbidity/severe underlying disease, severity of actual illness/futility and, prehospital disability.

The Institutional Ethics and Clinical Trials Committee approved the study protocol.

### 2.1. Statistical Analysis

Normal distribution of variables was assessed using the Kolmogorov-Smirnov test. Quantitative variables with normal distribution are expressed as mean ± S.D.; nonnormal distribution variables are shown as medians and interquartile ranges. Qualitative variables are shown as percentages.

Comparisons between patients accepted and patients refused to ICU and comparisons between patients admitted to ICU and patients refused to ICU admission because they were “too ill to benefit,” were based on the Student's *t*-test, Mann-Whitney test, and chi-square test, for quantitative variables with normal distribution, continuous variables with nonnormal distribution, and qualitative variables, respectively.

Level of statistical significance was set to *P* values less than 0.05 and results are expressed with their 95% confidence intervals.

Logistic regression models were built for multivariate analysis to identify factors associated with ICU refusal. Univariate analysis of main variables registered at evaluation time (age, sex, reason for triage, referral site, beds availability, BI [11], CRMS [12], CCI [13], APACHE II [14], and SOFA [15]) was done. Predictor variables that were statistically significant when evaluated individually against ICU refusal were included in the forward stepwise multiple logistic regression analysis. The results were presented as odds ratios with the appropriate 95% confidence interval.

Statistical analysis was performed using SPSS 18.0 software (SPSS Inc., Chicago, IL).

## 3. Results

During the study period 338 patients older than 75 years were evaluated for ICU admission. Four patients refused ICU care and were excluded from the analysis.

The request came from the emergency room (56.3%), medical or surgical wards (33.5%), and operating/recovery room (10.2%).

The baseline characteristics of all patients evaluated for ICU admission are shown in Table 1 and the main reason for ICU evaluation is shown in Table 2. There were statistical significant differences between the group of accepted versus rejected patients in respiratory, infectious, and renal/genitourinary reasons for the ICU triage decision. The percentage of patients triaged due to respiratory support was higher in the refused group of patients, whereas there was higher rate of patients with infectious reason for critical care on patients admitted to ICU.

**Figure 1:**
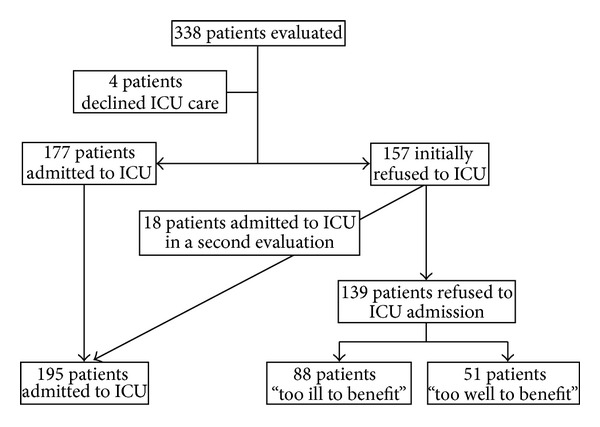
Flow chart of inclusion and exclusion patients.

Of 334 patients who underwent screening for eligibility, 177 patients were initially admitted to ICU (18 patients were transferred to another ICU in another hospital due to lack of available beds in our unit) and 18 patients refused for admission due to being “too well to benefit” were admitted to the ICU within 24 hours after a second evaluation. Thus, 195 patients were included in the study. Among the 139 patients refused to ICU admission, 51 patients (36.7%) were refused because they were “too well to benefit” and 88 patients (63.3%) because they were “too ill” (Figure 1). Causes for refusal of patients considered “too ill” were being “too old” in 54.5% of patients, high grade of comorbidity/severe underlying disease in 68.2%, severity of actual illness/futility in 51.1%, and prehospital disability in 37.5%. Two or more causes were present in 71.5% of patients.

Outcomes of patients who were candidates to ICU are shown in Table 3. Hospital mortality rate of the whole study cohort was 42.1%: 35.8% in the admitted group, 55.6% in the “too ill to benefit” group, and 5.9% in the 51 patients considered “too well to benefit.”

There was higher 1-year mortality among patients refused to ICU (71.3% versus 41.0% in patients admitted to ICU, *P* < 0.01) (Table 3). This 1-year mortality was higher than expected according to their CCI (median CCI [13] of 3 correlates with an expected 1-year mortality of 52%) [13].

We next investigated the differences between the baseline characteristics of patients admitted to ICU and of patients refused to ICU admission because they were “too ill to benefit.” Patients refused to ICU admission and “too ill to benefit” were older (mean age of 82 (79–85) years versus 81 (78–84) years in admitted to ICU patients, *P* = 0.012) and had more comorbidity (mean CCI [13] 3 (2–4) versus 1 (0–2) in patients admitted to ICU, *P* < 0.001), worse functional situation (mean BI [11] 67.50 (40–100) versus 100 (85–100) of patients admitted to ICU, *P* < 0.001), and worse prior mental status (none of patients admitted to ICU had a severe mental incapacity versus 5.6% in rejected patients, *P* = 0.004). Conversely, there was no difference in gender or severity of illness measured by SOFA [15] and APACHE II [14] scores between patients refused or admitted to ICU (Table 4).

To assess the impact of clinical variables associated with the decision to refuse ICU admission in “too ill to benefit” patients, we performed a logistic regression analysis. We identify that comorbidity according to the CCI [13], prior functional status measured by the Barthel Index [11], age, and no available ICU beds are factors independently associated with refusal of ICU admission (Table 5).

## 4. Discussion

In this prospective single-center study, we have found that, among patients older than 75 years, those refused to ICU admission have higher hospital mortality and 1-year mortality than patients admitted to ICU. Patient-related factors associated with refusal to ICU admission were more related to age, or prior functional or comorbidity status, than to the severity of illness at time of triage.

We observed that the rate of refusal of elderly patients (older than 75 years) to our ICU (41.62%) was slightly higher than the rate described in a previous study in patients older than 74 years (25%) [3]. However, this rate was lower than those found in two French studies with octogenarians patients, where the rate was 73% [9] and 88% [1], although a great variability between centers was observed. This finding may be influenced by the fact that 51 of 139 patients (36.69%) were considered “too well to benefit” or by the variation in hospital organization across countries.

Analysis of the hospital mortality of patients refused to ICU because they were “too well” or “too ill” to benefit revealed that hospital mortality of those patients was 5.9% and 56.3%, respectively. As expected, patients who were refused to admission due to futility had higher observed mortality than those admitted. In both groups of refused patients, we found lower hospital mortality than that described in previous studies. Garrouste-Orgeas et al. [9] found higher hospital mortality rates than in our study (17.6% in patients “too well” and 70.8% in those “too ill”), although their study only included octogenarians. Similar rates were reported in the study of Sprung et al. [3] in patients of 75 years old and older (20% in patients “too well” and 77.2% in those “too ill”), whereas Joynt et al. [17] described a hospital mortality of 90% in patients refused to ICU admission due to futility and of 8% in patients considered too well to benefit from ICU treatment.

We have an unexpected high rate of survival of patients refused to ICU due to being too ill (44%). In contrast, Joynt et al. described a low rate of hospital survival of 10% in patients refused to ICU because of perceived futility [17]. This is in agreement with the low relevance in our case of the severity of illness as variable for the refusal decision. These data highlight the difficulty of assessing futility for intensivists. The 1-year mortality rate in our study was 41% in admitted patients, which is lower than that described before in elderly patients (42–70%) [8, 9]. Patients refused to ICU due to being “too ill to benefit” had not only a higher hospital mortality, but also a higher mortality rate during 1-year followup as in previous studies [8, 9].

Trying to understand the reasons to adopt decisions of refusing ICU admission, we investigated the factors associated with futility. Factors associated with patients “too well to benefit” were not analyzed, because these patients were really inappropriate referrals. As in previous studies, we found that patients refused to ICU have a worse previous functional (BI [11]) and cognitive (Cruz Roja Mental Scale [12]) status, higher comorbidity (CCI [13]), but not higher severity of illness at time of triage (APACHE II [14] and SOFA [15] scores), compared with those accepted to ICU [1, 3, 7, 9]. We found that among all the patients referred for ICU admission, 20.1% of patients were dependent (defined as having a BI [11] lower than 60), but only 2.09% had a severe mental incapacity. There are few data about this point. Garrouste-Orgeas et al. [18] report that only 6.9% of adult patients referred to ICU admission were dependent at the time of triage. Daubin et al. [19] in their study about predictors of mortality and short-term physical and cognitive dependence in critically ill persons of 75 years and older admitted in ICU report that only 57% and 40% of patients were completely physically dependent measured by the Katz Index of Activity of Daily Living [20] and cognitive independent measured by individual components of the Lawton Index of Daily Living [21], respectively, and 1% and 7% were completely dependent. In our study, the 48.7% in the admitted group and 30.7% in the refused group were completely physically independent (defined as BI [11] 100); 11.3% and 15.9%, respectively, were completely physically dependent (defined as BI [11] lower than 20). Completely normal cognitive situation was found in 74.6% in the admitted group and in 46.0% in the refused group, whereas severe mental incapacity was found in 0% and 5.6% in the admitted and refused groups respectively.

We found that age, comorbidity, prior functional status, and full unit were independent variables associated with ICU refusal. There are few data about these points. Garrouste-Orgeas et al. found that age, medical status, and full unit were the factors associated with refusal in octagenarians patients [9]. Iapichino et al. found that age and more than one ICU triage were associated with ICU refusal in general population [7]. Joynt et al. found that age, diagnostic category, severity of illness, and operative factors were important factors in ICU refusal [17]. We thought that we did not have triage (the process of sorting referred patients in order of priority [22, 23]) as a reason for the decision, because if we thought that the patient needed admission to the ICU, the patient was transferred to another hospital. However, we found a strong effect of bed availability for refusing patients to ICU admission. This finding is almost always found by other authors [5, 18, 24, 25].

This study had several limitations. First, it was conducted in a single centre and our results may not be applicable to other hospitals or healthcare systems. Wide variability from one hospital to another has been described [1]. Second, it was observational and the lack of randomization is a source of inaccuracy. Third, we did not measure quality of life in survivors at one-year followup and self-dependency and quality of life may be poorer than in general population [9]. Our strengths are that it is a study with a prospective observational design, with a large enrolment period, realized exclusively in elderly patients (older than 75 years).

## 5. Conclusions

In conclusion, not only age, but also prior functional status measured by the Barthel Index [11] and the degree of comorbidity measured by the Charlson Comorbidity Index [13] can help us to make the right decision of admitting or refusing to ICU patients older than 75 years old. However, we need more multicenter and randomized studies to improve our ICU admission criteria.

## Supplementary Material

In the supplementary material we have shown the scales used in this study: The Barthel Index, Cruz Roja Mental Scale and The Charlson Comorbidity Index.

## Figures and Tables

**Table 1:** Clinical characteristics of the patients at first ICU.

Patients characteristics	All patients evaluated for ICU admission (*n* = 334)
Age*	81 (78–85)
	143 (42.8%)
81–85 years	126 (37.7%)
	65 (19.5%)
Gender: male	168 (50.3%)
Charlson Comorbidity Index*	2 (1–3)
Comorbidity	
Low	141 (42.5%)
Medium	90 (27.1%)
High	101 (30.4%)
Barthel Index*	95 (65–100)
Completely independent	156 (49.7%)
Mild dependence	95 (30.3%)
Moderate dependence	34 (10.8%)
Severe dependence	10 (3.2%)
Completely dependent	19 (6.1%)
Functional dependence (Barthel Index < 60)	63 (20.1%)
Cruz Roja Mental Scale	
Completely normal	205 (65.1%)
Light disorders of disorientation in time	66 (21%)
Disorientation in time	35 (11.1%)
Complete disorientation	2 (6%)
Complete mental disorder	3 (1%)
Dementia very evident	4 (1.3%)
Severe mental incapacity (CRMS > 3)	7 (2.09%)
APACHE II*	15 (11–22)
SOFA*	4 (1–7)

Data are shown as number (percent), except for those marked with *that are shown as median (P_25_–P_75_).

**Table 2:** Main reason for patient evaluation at first ICU triage.

	Accepted to ICU *n* = 195 (57.7%)	Refused to ICU *n* = 139 (42.3%)	*P* value accepted versus refused
Cardiac arrest	10 (5.1%)	10 (7.2%)	0.433
Cardiovascular	112 (57.4%)	72 (51.8%)	0.307
Respiratory	8 (4.1%)	22 (15.8%)	0.000
Neurologic	6 (3.1%)	9 (6.5%)	0.139
Intoxications	3 (1.5%)	4 (2.9%)	0.400
Digestive	4 (2.1%)	2 (1.4%)	0.678
Surgical	7 (3.6%)	2 (1.4%)	0.231
Infectious	43 (22.1%)	14 (10.1%)	0.004
Renal/genitourinary	0 (0%)	4 (2.9%)	0.017
Others	2 (1%)	0 (0%)	0.513

Data are shown as number (percentage).

**Table 3:** Outcomes of patients who were candidates to ICU.

	Patients admitted to ICU (*n* = 195)	Patients refused to ICU (*n* = 88)	*P* value
Hospital mortality	70/195 35.8%	49/88 55.6%	0.002
Length of hospital stay*	11 (6–21.5)	7.5 (2–12.25)	0.000
Mortality during 1-year followup	10/125 8.0%	13/38 34.2%	0.000
1-year mortality	80/195 41.0%	62/87 71.3%	0.000
% patients who were rehospitalized during 1-year followup	68/115 59.1%	17/38 44.7%	0.122

Data are shown as number (percent), except for those marked with *that are shown as median (P_25_–P_75_).

**Table 4:** Baseline characteristics of patients who were candidates to ICU.

	Patients admitted to UCI *n* = 195 (68.9%)	Patients refused to ICU *n* = 88 (31.0%)	*P* value
Age*	81 (78–84)	82 (79–85)	0.012
>75–80 years	93 (47.7%)	29 (33%)	
81–85 years	66 (33.8%)	38 (43.2%)	
>85	36 (18.5%)	21 (23.9%)	
Gender: male	97 (49.7%)	47 (53.4%)	0.568
Charlson Comorbidity Index*	1 (0–2)	3 (2–4)	0.000
Comorbidity			0.000
Low	101 (52.1%)	18 (20.5%)	
Medium	53 (27.3%)	24 (27.3%)	
High	40 (20.6%)	46 (52.3%)	
Barthel Index*	100.0 (85–100)	67.5 (40–100)	0.000
Completely independent	95 (48.7%)	27 (30.7%)	
Mild dependence	65 (33.3%)	23 (26.1%)	
Moderate dependence	11 (5.6%)	18 (20.5%)	
Severe dependence	2 (1%)	6 (6.8%)	
Completely dependent	22 (11.3%)	14 (15.9%)	
Functional dependence (Barthel Index < 60)	35 (17.9%)	38 (43.2%)	0.000
Cruz Roja Mental Scale			0.001
Completely normal	132 (74.6%)	40 (46.0%)	
Light disorders of disorientation in time	29 (16.4%)	28 (32.2%)	
Disorientation in time	16 (9.0%)	13 (14.9%)	
Complete disorientation	0 (0%)	2 (2.3%)	
Complete mental disorder	0 (0%)	3 (3.4%)	
Dementia very evident	0 (0%)	1 (1.1%)	
Severe mental incapacity (CRMS > 3)	0 (0%)	4 (5.6%)	0.004
APACHE II*	17 (11–23)	17 (11–24)	0.660
SOFA*	5 (1–8.25)	4 (2–6)	0.301

Data are shown as number (percentage), except for those marked with *that are shown as median (P_25_–P_75_).

**Table 5:** Factors associated with ICU refusal.

Univariate analysis		
	Odds ratio (95% CI)	*P* value
Age	1.07 (1.01–1.14)	0.011
Sex (male)	0.83 (0.52–1.43)	0.568
APACHE II	1.01 (0.98–1.04)	0.414
SOFA	0.93 (0.87–1.00)	0.056
Charlson Comorbidity Score	1.58 (1.33–1.89)	0.000
Barthel Index	0.97 (0.96–0.98)	0.000
Cruz Roja Mental Scale	2.12 (1.52–2.96)	0.000
No beds available	0.37 (1.13–0.99)	0.040
Referral site		
Reason for patient evaluation		
Cardiac arrest	2.37 (0.94–5.92)	0.061
Cardiovascular	0.48 (2.29–0.81)	0.001
Respiratory	7.32 (3.09–1.32)	0.000
Neurologic	2.72 (0.88–8.35)	0.079
Intoxications	3.05 (0.67–13.91)	0.150
Digestive	0.55 (0.06–4.98)	0.591
Surgical	0.31 (0.04–2.55)	0.270
Infectious	0.40 (0.18–0.87)	0.020

Multivariate analysis		
	Odds ratio (95% CI)	*P* value

Age	1.09 (1.01–1.17)	0.018
Charlson Comorbidity Index	1.58 (1.31–1.91)	0.000
Barthel Index	0.97 (0.96–0.98)	0.000
No beds available	0.26 (0.08–0.81)	0.021
