# Supplementary material for: Characteristics and Outcomes of Elderly Patients Refused to ICU
Source: ScientificWorldJournal. 2013 Dec 25;2013:590837. doi: 10.1155/2013/590837 (PMC3886377; doi:10.1155/2013/590837)
Supplement: Supplementary file 1 — In the supplementary material we have shown the scales used in this study: The Barthel Index, Cruz Roja Mental Scale and The Charlson Comorbidity Index. [file 590837.f1.doc]

**SUPPLEMENTARY MATERIAL**

**1. Barthel Index**

The Barthel Index (BI)[26] asseses the ability of patient to perform in an independent or dependent form, 10 daily activities as feeding, transferring, toileting, continence and dressing. The scale ranges from 100 (completely independent) to 0 (completely dependent). Establish a dependency according to their score in 5 levels: completely independent (score: 100), mild dependence (score: more than 60), moderate dependence (score: 40-55), severe dependence (score: 20-35) and completely dependent (lower than 20). We defined the cutting point 60 above to define independence[16]. (Appendix 1).

| Activity | Description | Score |
| --- | --- | --- |
| FEEDING | Unable | 0 |
|  | Needs help cutting, spreading butter, etc., or requires modified diet | 5 |
|  | Independent | 10 |
| BATHING | Dependent | 0 |
|  | Independent (or in shower) | 5 |
| GROOMING | Needs to help with personal care | 0 |
|  | Independent face/hair/teeth/shaving (implements provided) | 5 |
| DRESSING | Dependent | 0 |
|  | Needs help but can do about half unaided | 5 |
|  | Independent (including buttons, zips, laces, etc.) | 10 |
| BOWELS | Incontinent (or needs to be given enemas) | 0 |
|  | Occasional accident | 5 |
|  | Continent | 10 |
| BLADDER | Incontinent, or catheterized and unable to manage alone | 0 |
|  | Occasional accident | 5 |
|  | Continent | 10 |
| TOILET USE | Dependent | 0 |
|  | Needs some help, but can do something alone | 5 |
|  | Independent (on and off, dressing, wiping) | 10 |
| TRANSFERS (BED TO CHAIR AND BACK) | Unable, no sitting balance | 0 |
|  | Major help (one or two people, physical), can sit | 5 |
|  | Minor help (verbal or physical) | 10 |
|  | Independent | 15 |
| MOBILITY (ON LEVEL SURFACES) | Immobile or < 50 yards | 0 |
|  | Wheelchair independent, including corners, > 50 yards | 5 |
|  | Walks with help of one person (verbal or physical) > 50 yards | 10 |
|  | Independent (but may use any aid; for example, stick) > 50 yards | 15 |
| STAIRS | Unable | 0 |
|  | Needs help (verbal, physical, carrying aid) | 5 |
|  | Independent | 10 |
| TOTAL (0-100) | | |

**2. Cruz Roja Mental Scale**

Cruz Roja Mental Scale (CRMS)[27] is a simple and easy to use scale, the assessor must classify the patient in mental status that is closest to the current situation of patient according information given by the patient or carer. Evaluates mental disability on a scale of 6 degress ranging from 0 (normal) and 5 (total inability). The questionnaire has been validated in a Spanish population[28]. A punctuation over 3 is considered a severe mental incapacity and has been correlated with increased mortality in hospitalized elderly patiens[16]. (Appendix 2).

| Score | Description |
| --- | --- |
| 0 | Completely normal |
| 1 | Light disorders of disorientation in time. He/she correctly maintains a conversation. |
| 2 | Disorientation in time. The conversation with him/her is possible but not perfect. He/she knew people, but sometimes forget something. He/she has character disorders. He/she have occasional incontinence. |
| 3 | Complete disorientation. Impossible to maintain a logical conversation. He/she confuses people. He/she have clear mood disorders. He/she have frequent episodes of incontinente. |
| 4 | Complete mental disorder with clear disorientation. Habitual or total incontinence. |
| 5 | Dementia very evident. He/she do not know the people. Vegetative life with or without aggressiveness. Total incontinence. |

**3. Charlson Comorbidity Index**

The Charlson Comorbidity Index (CCI) [13] is the sum of comorbidities observed in patients, which have a weighted score based on the relative risk of 1-year mortality. We defined 3 levels of comorbidity according to the Charlson Comobity Index: low (score: 0 or 1), medium (score: 2) and high (score: 3 or over)[13]. (Appendix 3).

| Score | Condition |
| --- | --- |
| 1 | Myocardial infarct |
| 1 | Congestive heart failure |
| 1 | Peripheral vascular disease |
| 1 | Cerebrovascular disease |
| 1 | Dementia |
| 1 | Chronic pulmonary disease |
| 1 | Connective tissue disease |
| 1 | Ulcer disease |
| 1 | Mild liver disease |
| 1 | Diabetes |
| 2 | Hemiplegia |
| 2 | Moderate or severe renal disease |
| 2 | Diabetes with end organ damage |
| 2 | Any tumor |
| 2 | Leukemia |
| 2 | Lymphoma |
| 3 | Moderate or severe liver disease |
| 6 | Metastatic solid tumor |
| 6 | Acquired immunodeficiency syndrome |
